# Ultrasound assessment of malnutrition in infancy: a pilot case-control study

**DOI:** 10.1186/s12887-023-04479-z

**Published:** 2024-01-03

**Authors:** Meng-Fan Tai, Ruth Bvalani, Bazwell Nkhalema, Emmie Mbale, Karen Chetcuti, Pui-Ying Iroh Tam

**Affiliations:** 1 Paediatrics and Child Health Research Group, Malawi-Liverpool Wellcome Research Programme, Blantyre, Malawi; 2 Department of Paediatrics and Child Health, Queen Elizabeth Central Hospital, Blantyre, Malawi; 3 Department of Radiology, Queen Elizabeth Central Hospital, Blantyre, Malawi; 4 Kamuzu University of Health Sciences, Blantyre, Malawi; 5 Liverpool School of Tropical Medicine, Liverpool, UK; 6 Current affiliation: Department of Paediatrics, Taipei Tzu Chi Hospital, New Taipei City, Taiwan; 7 Malawi-Liverpool Wellcome Research Programme, Queen Elizabeth Central Hospital, Kamuzu University of Health Sciences, P.O. Box 30096, Chichiri, Blantyre 3, Malawi

**Keywords:** Ultrasonography, Malnutrition, Infant, Subcutaneous fat, Skeletal muscle

## Abstract

This study aimed at determining the intra- and inter-rater reliability in ultrasound body composition measurements and investigating the differences between malnourished and non-malnourished infants. Sonographic images for measurements of fat and muscle thickness were compared between 9 malnourished and 9 non-malnourished hospitalized infants. The mean of fat and muscle thickness sums were 12.44 ± 7.58 mm and 28.98 ± 7.18 mm, respectively. The intra- and inter-rater intraclass correlation coefficient were above 0.9 for both measurements, indicating high intra- and inter-rater reliability. Compared to non-malnourished infants, malnourished infants have 45% of fat thickness sum and 71% of muscle thickness sum. Ultrasound measurements of body composition in infants were different between hospitalized malnourished and non-malnourished infants. This approach has the potential to be utilized more broadly, from assessing the nutritional status of critically ill infants in intensive care units to screening for malnutrition in high-risk infant populations.

## Introduction

Malnutrition affects an estimated 45 million and 149 million children with wasting and stunting affected, respectively [1]. The reliability and predictive value of WHO guidelines for diagnosing malnutrition in infants have been questioned [2]. While the current identification method of malnutrition is anthropometry, body composition such as fat mass and fat-free mass is increasingly recognized as a more sensitive measure of growth and nutritional status [3]. The validated methods for body composition measurements in infants such as air displacement plethysmography and dual energy x-ray absorptiometry require heavy equipment and are expensive [4]. An alternative device is a portable ultrasound, which is non-invasive, easy to utilize at the patient’s bedside, and demonstrated to be safe with good intra- and inter-rater reliability in studies measuring body composition in adults [5]. The objective of this pilot study was to evaluate the feasibility of ultrasound in diagnosing malnutrition in infants, to determine the intra- and inter-rater reliability in ultrasound measurements, and to investigate differences in ultrasound measurements between malnourished and non-malnourished infants.

## Methods

We conducted a pilot case-control study at Queen Elizabeth Central Hospital in Blantyre, Malawi from October to December 2022. We recruited hospitalized, malnourished patients under six months old and an equal number of hospitalized, non-malnourished age- (within 30 days of age) and gender-matched controls. Malnutrition was defined as weight-for-age less than − 2 z-score [1, 2]. We excluded infants with gross limb asymmetry or gross abdominal anomaly, or those who were too clinically unstable to be transported to the ultrasound room for measurements.

We collected anthropometric measurements in duplicate by each examiner on the right side of the body in accordance with the International Standards for Anthropometric Measurements. Muscle and subcutaneous fat thickness measurement sites [6, 7] were marked on the right side of the body by one operator (MT) and double-checked by one of the other two operators (BN, RB). Prior to starting and during the examination, calm and natural sleep were promoted while infants were in a supine position. Each of the three operators measured each of the measurement sites by ultrasound in duplicate. Measurements were taken using a minimal probe compression technique by placing the transducer on a thick layer of ultrasound gel at a 90° angle to the site of interest. Transverse images were taken in duplicate by a commercial real-time ultrasound system (SonoSite X-PORTE, FUJIFILM SonoSite, Bothell, WA, USA) with a linear array transducer operated at 6 to 13 MHz (HFL38xp, FUJIFILM SonoSite, Bothell, WA, USA). Informed consent was obtained from all subjects and/or their legal guardian. The study was approved by the University of Malawi College of Medicine (P.03/22/3600) and the Liverpool School of Tropical Medicine (22–023) Research Ethics Committees.

To evaluate intra- and inter-rater reliability, the intraclass correlation coefficient (ICC) and 95% confidence interval were calculated based on a two-way random effects model with average measures and absolute agreement (values greater than 0.90 indicates excellent reliability), following guidelines for selecting and reporting intraclass correlation coefficients for reliability research [8]. We examined for normal distribution using the Shapiro-Wilk test. IBM SPSS version 29 (Armonk, NY) was used for all analyses.

## Results

Eighteen patients were included in the study (Table 1), and a total of 270 and 216 ultrasound images were used for the inter-rater reliability test of the fat and muscle thickness, respectively. None of the patients were dehydrated during the examination, and only one patient had oedematous limbs over the left side which did not affect our measurements.

**Table 1:** Patient characteristics

Characteristics	Malnourished infants (n = 9)	Non-malnourished infants (n = 9)
Patient demographics		
Age, months	2.90 ± 1.59	2.68 ± 1.43
Female, n (%)	4 (44)	4 (44)
Weight-for-age z-score	-3.88 ± 0.88	-0.01 ± 1.09
Weight-for-length z-score	-2.24 ± 2.28	0.09 ± 1.58
Length-for-age z-score	-2.50 ± 1.15	0.00 ± 1.18
Birth weight, kg	2.76 ± 0.93	3.01 ± 0.57
Prematurity, n (%)	2 (22)	1 (11)
Positive maternal HIV status, n (%)	1 (11)	4 (44)
Hydrocephalus, n (%)	3 (33)	3 (33)
Anthropometric measurements		
Weight, kg	3.52 ± 0.87	5.44 ± 1.72
Supine length, cm	53.64 ± 4.87	57.58 ± 5.26
Head circumference, cm	39.50 ± 3.22	42.10 ± 4.57
MUAC, cm	10.39 ± 1.15	13.17 ± 1.73
Waist circumference, cm	36.62 ± 3.65	41.62 ± 6.22
Sum of skinfold thickness, mm	20.18 ± 7.22	32.88 ± 7.45
Ultrasound measurements		
Fat thickness, mm		
Biceps brachialis	1.23 ± 1.07	3.43 ± 1.39
Quadriceps femoris	2.33 ± 1.77	5.42 ± 2.21
Anterior tibialis	1.69 ± 1.27	3.29 ± 1.63
Rectus abdominis	1.03 ± 1.02	2.29 ± 1.03
Midline of abdomen	1.03 ± 0.94	1.91 ± 0.88
**Sum of five sites**	**7.77 ± 5.49**	**17.12 ± 6.54**
Muscle thickness, mm		
Biceps brachialis	4.98 ± 1.41	8.14 ± 2.18
Quadriceps femoris	7.27 ± 1.10	1.184 ± 3.93
Anterior tibialis	7.31 ± 1.33	8.72 ± 1.76
Rectus abdominis	1.86 ± 0.28	2.6 ± 0.77
**Sum of four sites**	**24.34 ± 2.52**	**34.16 ± 7.03**

MUAC, mid-upper arm circumference

**Fig. 1:**
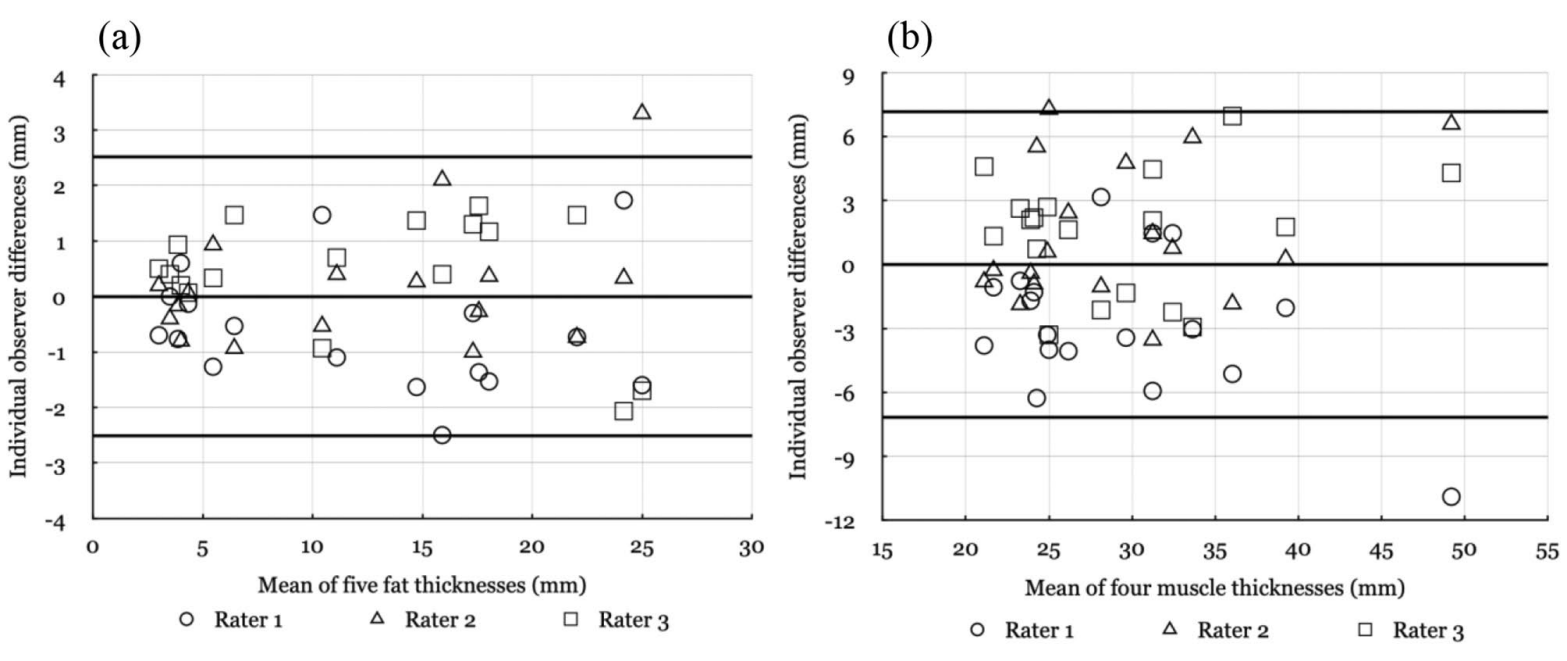
Modified Bland-Altman plot for (**a**) the sum of five fat measurements and (**b**) the sum of four muscle measurements among infants

The Pearson correlation coefficient for inter-rater reliability was 0.95 and 0.94 for fat and muscle measurements, respectively (Table 2). Modified Bland-Altman plots for (a) the sum of five fat measurements and (b) the sum of four muscle measurements with 95% limits of agreement were constructed to show the observer variability (Fig. 1) [9]. The fat thickness mean was 12.44 ± 7.58 mm with the standard deviation (SD) of observer differences being 1.28 mm and its 95% limits of agreements were between ± 2.51 mm. The muscle thickness mean was 28.98 ± 7.18 mm with SD of observer differences was 3.66 mm and its 95% limits of agreements were between ± 7.17 mm.

**Table 2:** Intra- and inter-rater reliability of muscle and fat ultrasound

	Fat thickness (n = 18*5 = 90)			Muscle thickness (n = 18*4 = 72)	
Intra-rater reliability	ICC (95% CI)	CoV		ICC (95% CI)	CoV
Rater 1	0.973 (0.959–0.982)	2.44%		0.976 (0.961–0.985)	6.10%
Rater 2	0.967 (0.950–0.978)	2.66%		0.975 (0.959–0.984)	3.84%
Rater 3	0.981 (0.971–0.988)	1.00%		0.994 (0.991–0.996)	1.20%
Inter-rater reliability	ICC (95% CI)	SEE (mm)		ICC (95% CI)	SEE (mm)
	0.954 (0.934–0.968)	0.58		0.929 (0.889–0.955)	1.46

CoV, coefficient of variation; ICC, intraclass correlation coefficient; SEE, standard error of the estimate

**Fig. 2:**
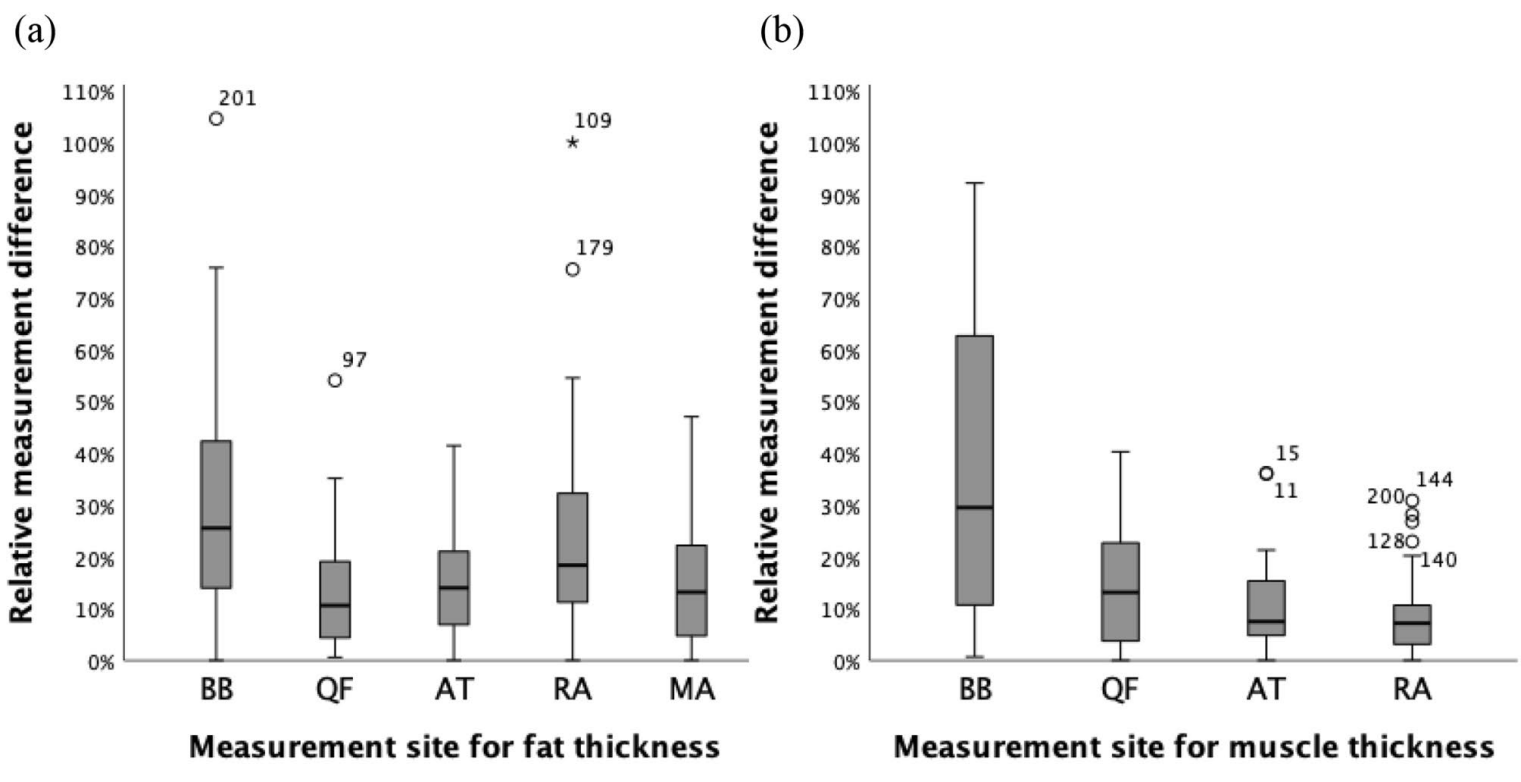
Relative measurement difference in (**a**) fat and (**b**) muscle thickness at each measurement site (n=54). BB, biceps brachialis; QF, quadriceps femoris; AT, anterior tibialis; RA, rectus abdominis; MA, midline abdomen

**Fig. 3:**
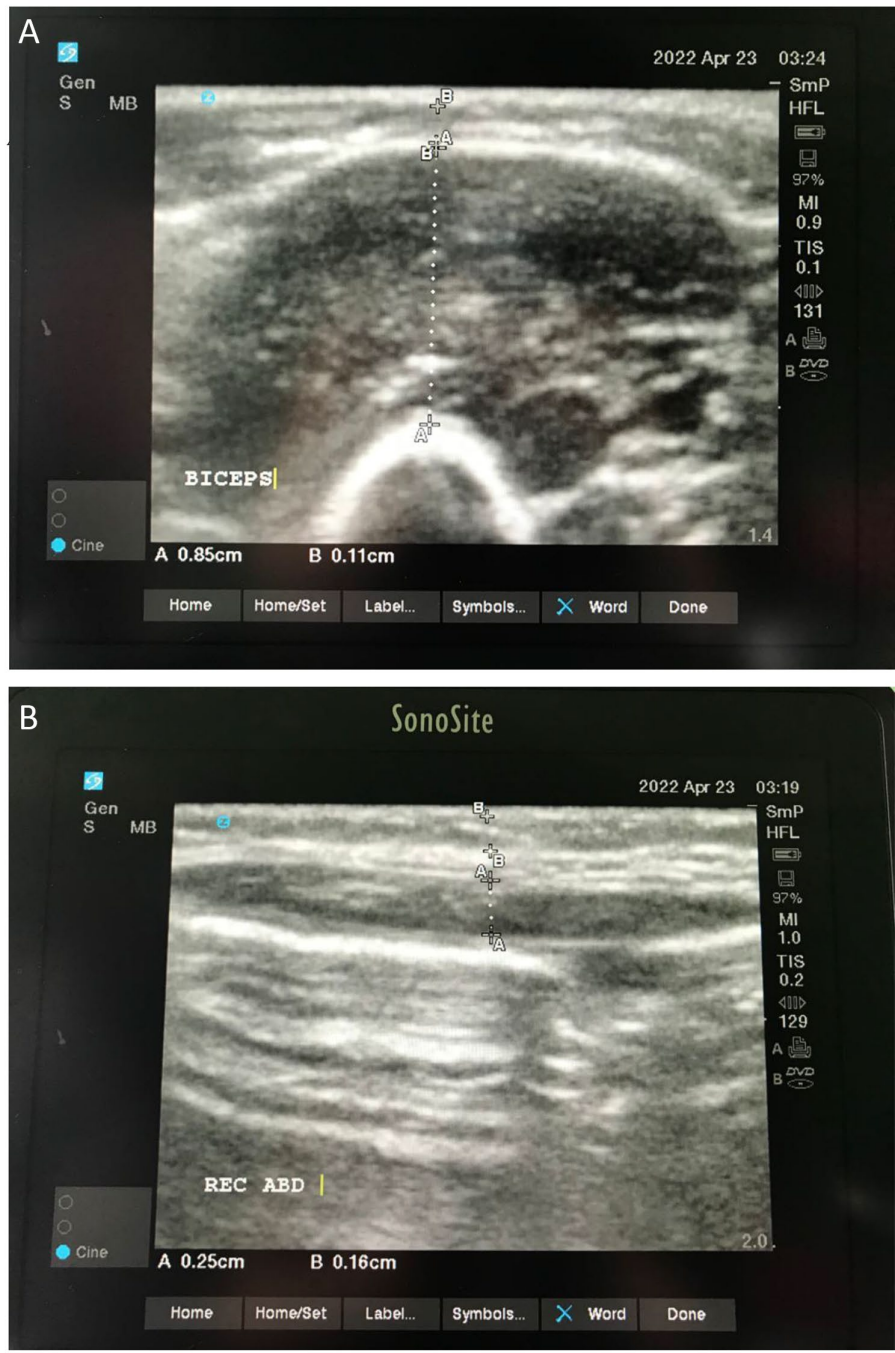
Ultrasound of (**A**) biceps brachialis; (**B**) rectus abdominis

The median of absolute measurement differences (ABS) in the sum of five fat measurements was 0.78 mm, which was 6.29% of mean fat thickness sums. The median ABS in the sum of four muscle measurements was 2.53 mm, i.e. 8.74% of mean muscle thickness sums. Figure 2 shows the relative measurement difference (REL) from their mean in (a) fat and (b) muscle thickness at each measurement site (n = 3*18 = 54). The medians of ABS in fat thickness ranged from 0.17 to 0.48 mm at individual measurement site with the medians of REL as 25.54%, 10.63%, 13.99%, 18.35%, and 13.18% at biceps, quadriceps, anterior tibialis, rectus abdominis, and midline abdomen, respectively (Fig. 3). For muscle thickness, the medians of ABS ranged from 0.13 to 2.00 mm. The medians of REL in muscle thickness were 29.49%, 13.15%, 7.51%, and 7.21% at biceps, quadriceps, anterior tibialis, and rectus abdominis, respectively.

The sum of fat thickness and the sum of muscle thickness in the malnourished infants were about 45% and 71% of the values in the non-malnourished infants, respectively (Table 1). Measurements over biceps brachialis had the largest differences between the two groups (36% and 62% of the values in non-malnourished infants) among all measurement sites, followed by measurements over quadriceps.

## Discussion

This proof-of-concept study conducted in freely-movable infants under six months old in a resource-limited setting demonstrates that ultrasound measurement of body composition (fat and muscle) is feasible, and has good intra- and inter-rater reliability. Previous research about fat and muscle ultrasound for infants was mainly conducted among neonates or sedated patients in intensive care units [6, 7, 10].

Compared to older age groups, the intra- and inter-rater reliability were less optimal in infants aged under six months, compared to previous studies [10–12]. This may be because infants are not able to obey orders and have thinner layers of fat and muscle. The reliability of measurements may be improved by adequate practice for avoiding compression errors such as ensuring the ultrasound gel is thick and can be seen as a dark band in the ultrasound window [10, 11]. Other methods for improving the reliability of ultrasound include using software for identifying fat and muscle tissues [11].

Measurements over biceps brachialis had the largest differences between malnourished and non-malnourished infants, but they also had the largest REL among all measurement sites. In comparison, quadriceps may be a more promising choice for evaluating nutritional status, for having a smaller inter-rater variability and a larger difference among the two groups.

This study was limited by the small sample size, which was due to resource constraints. There is also intra- and inter-observer variability in technique with use of the ultrasounds, but we attempted to minimize the variability by having the same three operators collect measurements for all participants. Furthermore, our control subjects did not have severe acute illness, but their condition relating to their hospitalization could have affected their measurements, which we did not evaluate beyond anthropometry. Despite these limitations, however, our proof-of-concept pilot study revealed that the decrease in fat thickness was more marked in malnourished compared to non-malnourished infants. This study demonstrates the potential for ultrasound body composition to facilitate the early identification of at-risk infants.

## Data Availability

The datasets used and/or analysed during the current study available from the corresponding author on reasonable request.
